# DNA damage repair system in C57BL/6 J mice is evolutionarily stable

**DOI:** 10.1186/s12864-021-07983-7

**Published:** 2021-09-17

**Authors:** Xiaoyu Wang, San Ming Wang

**Affiliations:** Cancer Centre and Institute of Translational Medicine, Faculty of Health Sciences, University of Macau, Taipa, Macau

**Keywords:** *Brca1*, *Brca2*, DNA damage repair, C57BL/6 J, Variation, Evolution selection

## Abstract

**Background:**

DNA damage repair (DDR) system is vital in maintaining genome stability and survival. DDR consists of over 160 genes in 7 different pathways to repair specific type of DNA damage caused by external and internal damaging factors. The functional importance of DDR system implies that evolution could play important roles in maintaining its functional intactness to perform its function. Indeed, it has been observed that positive selection is present in *BRCA1* and *BRCA2 (BRCA)*, which are key genes in homologous recombination pathway of DDR system, in the humans and its close relatives of chimpanzee and bonobos. Efforts have been made to investigate whether the same selection could exist for *BRCA* in other mammals but found no evidence so far. However, as most of the studies in non-human mammals analyzed only a single or few individuals in the studied species, the observation may not reflect the true status in the given species. Furthermore, few studies have studied evolution selection in other DDR genes except *BRCA*. In current study, we used laboratory mouse C57BL/6 J as a model to address evolution selection on DDR genes in non-primate mammals by dynamically monitoring genetic variation across 30 generations in C57BL/6 J.

**Results:**

Using exome sequencing, we collected coding sequences of 169 DDR genes from 44 C57BL/6 J individual genomes in 2018. We compared the coding sequences with the mouse reference genome sequences derived from 1998 C57BL/6 J DNA, and with the mouse Eve6B reference genome sequences derived from 2003 C57BL/6 J DNA, covering 30 generations of C57BL/6 J from 1998 to 2018. We didn’t identify meaningful coding variation in either *Brca1* or *Brca2*, or in 167 other DDR genes across the 30 generations. In the meantime, we did identify 812 coding variants in 116 non-DNA damage repair genes during the same period, which served as a quality control to validate the reliability of our analytic pipeline and the negative results in DDR genes.

**Conclusions:**

DDR genes in laboratory mouse strain C57BL/6 J were not under positive selection across its 30-generation period, highlighting the possibility that DDR system in rodents could be evolutionarily stable.

**Supplementary Information:**

The online version contains supplementary material available at 10.1186/s12864-021-07983-7.

## Background

A genome is constantly damaged by internal metabolic factors and external environmental factors. In order to maintain genome stability, living organisms are equipped with a highly sophisticated DNA damage repair (DDR) system to effectively repair the damages. The DDR system is composed of multiple pathways including homologous recombination (HR), non-homologous end joining (NHEJ), Fanconi anemia pathway (FA), base excision repair (BER), nucleotide excision repair (NER), mismatch repair (MMR), and single-strand annealing (SSA). Each pathway consists of a group of genes to repair a specific type of DNA damage through their collaborative action.

As DNA damage repair is vital for survival, it would be expected that evolution selection play roles in maintaining a highly functional DNA damage repair machinery for survival and better fitness. *BRCA1* and *BRCA2* (*BRCA*) are two important DDR genes for repairing DNA double-strand break through homology recombination (HR) pathway and mutation in *BRCA* substantially increases cancer risk [1, 2]. Studies indeed revealed that *BRCA* in the humans and its close relatives of chimpanzee and bonobos are under positive selection [3]. However, the same type of selection was not observed in other mammals [4–9]. This raises the possibility that the same DNA damage repair genes in different species could be under different evolution selections [10]. Except a few cases, however, nearly all *BRCA* variation data reported from non-human mammals were derived from a single individual in the tested species. From population genetics point of view, it is questionable if the observation made in a single individual could represent the situation in the tested species. Further, few other DDR genes except *BRCA* have ever been analyzed for their evolution selection (Table 1). Therefore, it remains unclear for the relationship between DDR system and evolution selection, a fundamental question in biology for the mechanisms of genome stability maintenance.

**Table 1:** Previous evolutionary studies in *BRCA1* and *BRCA2* in mammals

Species	Tested case	Reference
Orangutan	1	[3]
Gorilla	1	
Chimpanzee	1	
Macaque	1	
Howler monkey	1	
Bush baby	1	
Flying Lemur	1	
Mouse	1	
Rat	1	
Chimpanzee	1	[6]
Gorilla	1	
Orangutan	1	
Rhesus Monkey	1	
Red Howler Monkey	1	
Greater Galago	1	
Flying Lemur	1	
Large Tree Shrew	1	
Cape Hare	1	
Cape Porcupine	1	
Spring Hare	1	
Mountain Beaver	1	
Eastern Fox Squirrel	1	
Southern Flying Squirrel	1	
Woodland Dormouse	1	
Coues’ Rice Rat	1	
Shaw’s Jird	1	
House Mouse	1	
Brown Rat	1	
Eastern Mole	1	
European Hedgehog	1	
Daubenton’s Bat	1	
Brazilian Free-tailed Bat	1	
Tomb Bat	1	
Spix’s Round-eared Bat	1	
Greater False Vampire Bat	1	
Solomons Flying Fox	1	
Roundleaf Bat	1	
Short-nosed Fruit Bat	1	
Horse	1	
Black Rhinoceros	1	
Pangolin	1	
Dog	1	
Cat	1	
Pig	1	
Llama	1	
Cow	1	
Hippopotamus	1	
Humpback Whale	1	
Sperm Whale	1	
Southern Tamandua	1	
Three-toed Sloth	1	
Nine-banded Armadillo	1	
Large Hairy Armadillo	1	
African Elephant	1	
Asiatic Elephant	1	
Dugong	1	
West Indian Manatee	1	
Rock Hyrax	1	
Western Tree Hyrax	1	
Aardvark	1	
Tailless Tenrec	1	
Lesser Hedgehog-tenrec	1	
Elephant Shrew	2	
Golden Mole	1	
Coarse-haired Wombat	1	
Chicken	1	
Frog	1	
Red kangaroo	2	[11]
Tree kangaroo	1	
Wallaroo	1	
Coarse-haired wombat	1	
Brush-tailed phasogale	1	
Long-nosed bandicoot	1	
Virginia opossum	1	
Silky shrew opossum	1	
Chicken	1	
Frog	1	
Chimpanzee	1	[5]
Gorilla	1	
Orangutan	1	
Rhesus Macaque	1	
Chimpanzee	2	
Gibbon	2	
Baboon	2	
Tamarin	1	
Owl monkey	1	
Mouse	1	
Rat	1	
Opossums	2	[12]
Chimpanzee	1	
Gorilla	1	
Orangutan	1	
Rhesus Macaque	1	
Mouse	1	
Dog	1	
Cow	1	
Chicken	1	
Xenopus	1	
Tetraodon	1	
Chimpanzee	1	[13]
Gorilla	1	
Orangutan	1	
Macaque	1	
Cow	1	
Dog	1	
Mouse	1	
Rat	1	
Chimpanzee	1	[14]
Orangutan	1	
Macaque	1	
Gorilla	1	
Mouse	1	
Cow	1	
Opossum	1	
Dog	1	
Chimpanzee	44	[4]
Rhesus macaque	44	
Bonobo	7	
Borneo Orangutan	1	
Agile Gibbon	1	
White-handed Gibbon	1	
Pileated Gibbon	1	
Siamang	1	
White-cheeked Gibbon	1	
Redcheeked Gibbon	1	
Crab-eating Macaque	1	
Olive Baboon	1	
Black Mangabey	1	
Wolf’s Guenon	1	
Talapoin	1	
Leaf Monkey	1	
Colobus	1	
Squirrel Monkey	1	
Howler Monkey	1	
Titi Monkey	1	
Golden mole	1	[7]
hedgehog	1	
Otter shrew	1	
Tenrec	1	
Dog	1	
Cat	1	
Aardwolf	1	
Cow	1	
Giraffe	1	
Pygmy hippo	1	
Hippopotamus	1	
Llama	1	
Humpback whale	1	
White-tailed deer	1	
Sperm whale	1	
River dolphin	1	
Pig	1	
Desert bat	1	
Short-nosed fruit bat	1	
Common vampire bat	1	
Old world sheath-tailed bat	1	
Old world leaf-nosed bat	1	
Asian false vampire bat	1	
Little brown bat	1	
Funnel-eared bat	1	
Fisherman bat	1	
Guinean slit-faced bat	1	
African slit-faced bat	1	
Tube-nosed fruit bat	1	
Flying fox	1	
Horseshoe bat	1	
Long-tailed bat	1	
Little yellow bat	1	
Rousette fruit bat	1	
Free-tailed bat	1	
Tomb bat	1	
Round-eared bat	1	
Malayan flying lemur	1	
Phillipine flying lemur	1	
White-toothed shrew	1	
European hedgehog	1	
Pyrenean desman	1	
Gymnure	1	
Eastern mole	1	
Long-tailed shrew	1	
European mole	1	
Chinese shrew mole	1	
Tree hyrax	1	
Rock hyrax	1	
Snowshoe hare	1	
Pika	1	
Old world rabbit	1	
Long-eared elephant shrew	1	
Checkered elephant shrew	1	
Black rhino	1	
Horse	1	
Tapir	1	
Pangolin	1	
Howler monkey	1	
Spider monkey	1	
Gorilla	1	
Gibbon	1	
Lemur	1	
Macacaque	1	
Galago	1	
Chimpanzee	1	
Orangutan	1	
Tarsier	1	
Asian elephant	1	
African elephant	1	
Mountain beaver	1	
American beaver	1	
Gundi	1	
Pacarana	1	
North American porcupine	1	
Pocket gopher	1	
Flying squirrel	1	
African dormouse	1	
Naked mole rat	1	
Cape porcupine	1	
Jumping mouse	1	
Jird	1	
Mouse	1	
Rice rat	1	
Spring hare	1	
Pocket mouse	1	
Dassie rat	1	
Brown rat	1	
Tree squirrel	1	
Blind mole rat	1	
Common tree shrew	1	
Large tree shrew	1	
Dugong	1	
Manatee	1	
Aardvark	1	
Three-toed	1	
Naked-tailed armadillo	1	
Hairy armadillo	1	
Two-toed sloth	1	
Silky anteater	1	
Long-nosed armadillo	1	
Nine-banded armadillo	1	
Six-banded armadillo	1	
Giant anteater	1	
Giant armadillo	1	
Lesser anteater	1	
Three-banded armadillo	1	
Pichi	1	
Quoll	1	
Brush-tailed marsupial mouse	1	
Planigale	1	
Woolly opossum	1	
Common opossum	1	
Thick-tailed opossum	1	
Short-tailed opossum	1	
Short-nosed rat kangaroo	1	
Tree kangaroo	1	
Musky rat-kangaroo	1	
Red kangaroo	1	
Greater glider	1	
Cuscus	1	
Koala	1	
Ring-tailed possum	1	
Wombat	1	
Monito del monte	1	
Marsupial mole	1	
Silky shrew opossum	1	
Chilean shrew opossum	1	
New Guinean spiny bandicoot	1	
Short-nosed bandicoot	1	
Long-nosed bandicoot	1	

**Fig. 1:**
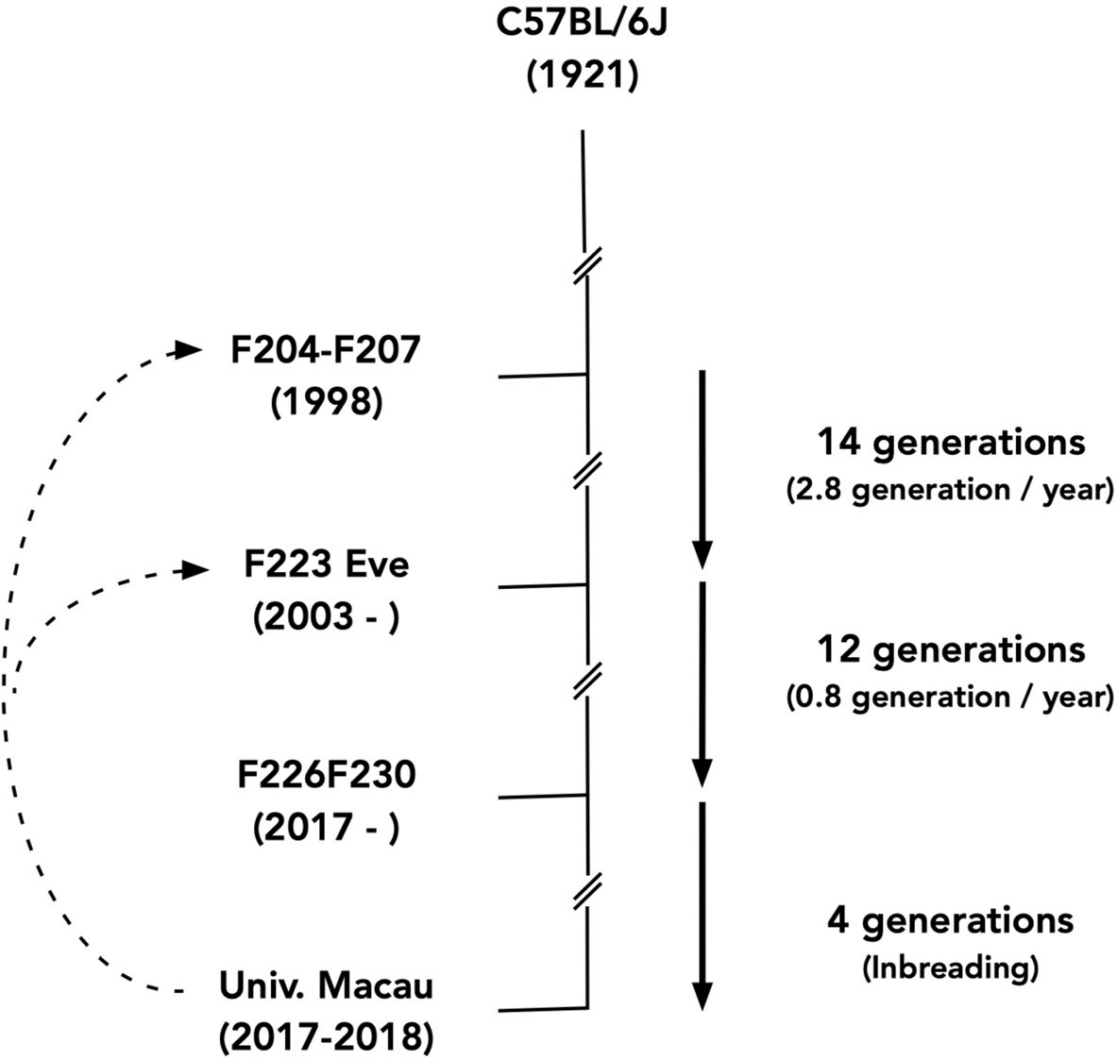
Origin and generations of C57BL/6 J. The C57BL/6 J was originated in 1921. Its genome in 1998 was sequenced by the Mouse Genome Project to develop the mouse genome reference sequences. After 14 generations, its genome in 2003 was sequenced to develop Eve6B genome sequences. The DNA used in current study was derived from 2018 C57BL/6 J, 30 generations after its genome was sequenced in 1998. See reference [17] for more details

Dynamic monitoring of genetic variation is a powerful approach to study evolution selection. This is best exemplified by the variation studies in *E. coli* by following its constant growth for four decades of over 60,000 generations under laboratory cultural conditions [15], and in laboratory rat by following its genetic variation in the genes involving in learning, circadian rhythm, and metabolism [16]. C57BL/6 J is one of the most used laboratory mouse models in biological and oncogenic studies. C57BL/6 J is the descendent of cryopreserved embryo stock with clear genetic background (Fig. 1). Its DNA extracted in 1998 was used for the Mouse Genome Project to generate the mouse genome reference sequences [18], and its DNA extracted in 2003 was sequenced again to generate the mouse genome reference sequences B6Eve [17]. From 1998 and 2018, C57BL/6 J has passed 30 generations. We hypothesized that this period can be longer enough as an excellent model to test evolution selection in DDR system in C57BL/6 J, and the information could be helpful to understand evolution selection on DDR system in rodents as represented by C57BL/6 J.

In present study, we sequenced the coding region of C57BL/6 J genome using the DNA collected from 44 C57BL/6 J individuals in 2018. We searched the variants arisen after 1998 by comparing the mouse genome reference sequences derived from 1998 C57BL/6 J DNA and mouse genome reference sequences B6Eve derived from 2003 C57BL/6 J DNA. We found no evidence for genetic variation arisen in the 169 DDR genes including *Brca1* and *Brca2* during this period, while we did identify the genetic variation in 116 non-DDR genes involved in other functional categories. From the data, we conclude that DDR system in C57BL/6 J is evolutionarily stable during its 30-generation period.

## Results

### Identifying genetic variants

C57BL/6 J genome in 1998 was sequenced by the Mouse Genome Project to generate the mouse genome reference sequences. Since then, C57BL/6 J mice has been inbreeded for 30 generations (24 in Jackson Laboratory and 4 in University of Macau Animal Facility) by 2018 (14, Fig. 1). We collected genomic DNA in 2018 from 44 C57BL/6 J mice and performed exome sequencing and called coding variants. We applied the following procedures to ensure the accuracy for the variants called from the exome sequences: 1) Only the variants present in > 50% (22 individuals) of the mice were kept for further analysis; 2) Using both mouse genome reference sequences mm7 and mm10 assemblies as the references for variant calling; 3) use B6Eve variants as the third reference; 4) Using Sanger sequencing to validate the called variants. From the exome sequences collected in the 2018 C57BL/6 J DNA, we identified a total of 3024 variants (Supplementary Table 1), of which 883 (29.2%) were singleton, 1329 (43.9%) were between 2 and 21, and 812 (26.9%) were present in at least 22 mice and used for further analysis (Supplementary Table 2). We reasoned that by setting up this high bar, we can address better population variation rather than individual variation.

### Variants in DDR genes

We searched the 812 variants but didn’t identify the variants in *Brca1* and *Brca2*. We further searched the variants in the rest of 167 DDR genes involved in 7 DNA damage repair pathways but didn’t identify any variants in these genes neither (Supplementary Table 3A, B).

### Variants in non-DDR genes

We then annotated the 812 variants and identified 116 non-DDR genes with these variants, of which *Mroh2a*, a HEAT-domain-containing protein with unknown function, had the highest number of 85 variants, and c4b, a component in Complementary system, had the 2nd highest number of 53 variants (Table 3, Supplementary Table 4). We used Sanger sequencing to validate a set of the variants in the original 2018 DNA samples used in exome sequencing. Of the 15 variants tested, 10 (67%) were validated (Supplementary Table 5). The variants identified in the non-DDR genes provided the internal control in ensuring that the absence of variation in DDR genes were a true biological phenomenon instead of missed identification due possibly to technical errors.

## Discussion

C57BL/6 J genome in 1998 was sequenced to generate the mouse genome reference sequences. After 20 years from 1998 to 2018 covering 30 generations, we re-sequenced the coding genes of C57BL/6 J in 44 individuals in order to determine if there could be variation arisen during this period in the DDR genes in C57BL/6 J genome. Our study didn’t identify new variants in DDR genes including *Brca1* and *Brca2* in the C57BL/6 J genome. The presence of new variants in over a hundred of non-DDR genes during the same period provided a strong assurance for the reliability of the observed lack of selection in DDR genes, and ruled out the possibility that the lack of variation in the full set of DDR genes was due to technical failure. The data from our study indicate the absence of positive selection in DDR genes in C57BL/6 J during the 30-generation period.

The lack of positive selection in DDR genes is unlikely due to the short period of C57BL/6 J under investigation. The 20-years of 30 generations in C57BL/6 J is equivalent to 800 years in the humans when counting 1 year in mouse equals to 30-years in the humans per generation [19]. Studies showed that many *BRCA* variations in the humans occurred in recent human history. For example, 185delAG in *BRCA1*, a founder variant in Ashkenazi Jews population, was arisen around 750–1500 years ago [20]; 1499insA in *BRCA1*, a founder variant in Tuscany of Italy, was originated 750 years ago [21]; *BRCA1* c.5266dupC, another founder variant in Ashkenazi Jews population, was originated 1800 year ago [22].

Possibility exists that animal under long-term protected laboratory environment could experience relaxed selection pressure, leading to altered genetic variation [23]. If the time period is longer enough and the starting genome sequences are available, testing genetic variation in wild mice would determine if such possibility could exist for the observation made in C57BL/6 J in our study.

The reference genome sequences used can have impact on the variation identification. After mouse genome project accomplished in 2001, 10 different versions of C57BL/6 J genome reference sequences were generated, including the first version of mm1 released in 2010 to mm10 released in 2011, before the mm39 released in 2020 (https://genome.ucsc.edu/FAQ/FAQreleases.html). The different versions of the mouse genome reference sequences used basically the same raw sequence data generated by the mouse genome project, but the variation data between different version were substantially different, which unlikely reflects true variation but annotation artifacts. As such, using all different versions as the reference for variant identification could lead to high complexity and data inconsistence, and decrease reliability of the resulting variation data. On the other hand, using a single version of reference sequences for variant identification could miss potential variants not identifiable in the single version. To address the concerns, we used two later versions of mouse genome reference sequences, mm7 and mm10, as the references for variant identification; we also used the variation data from Eve B6 genome sequences derived from 2003 C57BL/6 J DNA as another reference; we further used Sanger sequencing to validate selected variants. The combinational use of these approaches in our study ensured reliability and sensibility of the variants identified from our study to address the issue of evolution selection in DDR system in C57BL/6 J.

The evidence for the presence of positive selection in DDR genes is mainly from *BRCA* in human, chimpanzee and bonobos [3]. We propose explanations for why positive selection in *BRCA* exists in humans and its close relatives, but not in other mammals as represented in laboratory mouse C57BL/6 J: The basic function of *BRCA* is to repair DNA double-strand break in order to maintain genome stability in mammals. Like many genes involving in essential biological function, *BRCA* must be maintained in stable condition to perform their essential work [24]. During evolution process*,* however, *BRCA* in humans, chimpanzee and bonobos acquired new function such as enhancing intelligent development [25], gene expression regulation [26], and reproduction [27] etc. Positive selection on these function is beneficial for better fitness; whereas *BRCA* in other mammals retains the classical function of DNA damage repair, therefore, maintains high stability in order to keep genome stability. The explanations may also be applicable to other DDR genes. It will be interesting to find more evidence to support these explanations in different mouse strains and different species.

## Conclusion

DDR genes in laboratory mouse strain C57BL/6 J were not under positive selection across its 30-generation period, highlighting the possibility that DDR system in rodents could be evolutionarily stable.

## Methods

### Sample source

C57BL/6 J mice used in this study was purchased from Jackson Laboratory in 2017, and inter-bred 4 generations in University of Macau Animal Facility. Mouse genomic DNA in 2018 was extracted from the tails of 44 C57BL/6 J mice (15 male and 29 female) using DNeasy Blood & Tissue Kit (Qiagen) following the instruction. The study was approved by University of Macau Animal Welfare Committee (UMARE-041-2017), and was carried out in accordance with relevant guidelines and regulations.

### Exome sequence, mapping and variant call

Exome sequencing was performed at pair-end (2 × 150) and > 100x in Illumina Hiseq 2500 through Novogen customer service (Novogen**,** Hong Kong). Sequences were aligned to mouse reference genome sequence mm7 and mm10 using BWA 0.7.17MEM module and rearranged by Samtools v1.9 with sort option. Duplicates were removed by Picard in Genome Analysis Tool Kit (GATK) v4.1.1.0. IndelRealinger, BaseRecalibrator and ApplyBQSR options in GATK were used for BAM data processing. GenotypeGVCFs in GATK was used to call variants from BAM files, and Annovar was used for annotation, 20% variant allele frequency was used as the cutoff for variant calling. CrossMap was used to convert mm7 identified variants into mm10 to generate a mm10-based single set of variants. The Eve6B variants contain 2652 coding-variants identified from the 2003 C57BL/6 J genome, which differed from the 1998 C57BL/6 J-based mouse genome reference sequence GRCm38 (Supplementary Table 6). The 3 variants of chr11: 3186080 G > A, chr11: 3187266 C > T, and chr11: 3187367 T > C in Sfi1 were eliminated from the mapping analysis as they were determined by B6Eve study as artifacts [17].

### Source of DNA damage repair genes

DNA damage repair-related genes were downloaded from KEGG DNA repair related pathways (http://software.broadinstitute.org/gsea/msigdb), which consists of 169 genes in 7 pathways of base excision repair (BER), DNA replication (DR), Fanconi anemia (FA), homologous recombination (HR), non-homologous end-joining (NHEJ), mismatch repair (MMR), and nucleotide excision repair (NER) (Table 2).

**Table 2:** List of DDR genes included in the study

Nucleotide Excision Repair	Homologous recombination	Fanconi anemia pathway	Non-Homologous End-Joining
*Ccnh*	*Atm*	*Atr*	*Dclre1c*
*Cdk7*	*Babam1*	*Atrip*	*Dntt*
*Cetn2*	*Bard1*	*Blm*	*Fen1*
*Cul4a*	*Blm*	*Brca1*	*Lig4*
*Cul4b*	*Brca1*	*Brca2*	*Loc731751*
*Ddb1*	*Brca2*	*Brip1*	*Mre11a*
*Ddb2*	*Eme1*	*Cenps*	*Nhej1*
*Ercc1*	*Mre11a*	*Cenps-Cort*	*Poll*
*Ercc2*	*Mus81*	*Cenpx*	*Polm*
*Ercc3*	*Nbn*	*Eme1*	*Prkdc*
*Ercc4*	*Pold1*	*Eme2*	*Rad50*
*Ercc5*	*Pold2*	*Ercc1*	*Xrcc4*
*Ercc6*	*Pold3*	*Ercc4*	*Xrcc5*
*Ercc8*	*Pold4*	*Faap100*	*Xrcc6*
*Gtf2h1*	*Rad50*	*Faap24*	**Dna Replication**
*Gtf2h2*	*Rad51*	*Fan1*	*Dna2*
*Gtf2h3*	*Rad51b*	*Fanca*	*Fen1*
*Gtf2h4*	*Rad51c*	*Fancb*	*Lig1*
*Gtf2h5*	*Rad51d*	*Fancc*	*Mcm2*
*Lig1*	*Rad52*	*Fancd2*	*Mcm3*
*Mnat1*	*Rad54b*	*Fance*	*Mcm4*
*Pcna*	*Rad54l*	*Fancf*	*Mcm5*
*Pold1*	*Rbbp8*	*Fancg*	*Mcm6*
*Pold2*	*Rpa1*	*Fanci*	*Mcm7*
*Pold3*	*Rpa2*	*Fancl*	*Pcna*
*Pold4*	*Rpa3*	*Fancm*	*Pola1*
*Pole*	*Rpa4*	*Hes1*	*Pola2*
*Pole2*	*Shfm1*	*Mlh1*	*Pold1*
*Bivm-Ercc5*	*Ssbp1*	*Mus81*	*Pold2*
*Gtf2h2c*	*Sycp3*	*Palb2*	*Pold3*
*Rad23a*	*Top3a*	*Pms2*	*Pold4*
*Rad23b*	*Top3b*	*Polh*	*Pole*
*Rbx1*	*Topbp1*	Poli	*Pole2*
*Xpa*	*Uimc1*	Polk	*Pole3*
*Xpc*	*Xrcc2*	Poln	*Pole4*
**Base Excision Repair**	*Xrcc3*	Rad51	*Prim1*
*Apex1*	**Mismatch Repair**	Rad51c	*Prim2*
*Apex2*	*Exo1*	Rev1	*Rfc1*
*Fen1*	*Lig1*	Rev3l	*Rfc2*
*Hmgb1*	*Mlh1*	Rmi1	*Rfc3*
*Hmgb1p1*	*Mlh3*	Rmi2	*Rfc4*
*Hmgb1p40*	*Msh2*	Rpa1	*Rfc5*
*Lig1*	*Msh3*	Rpa2	*Rnaseh1*
*Lig3*	*Msh6*	Rpa3	*Rnaseh2a*
*Mbd4*	*Pcna*	Rpa4	*Rnaseh2b*
*Mpg*	*Pms2*	Slx1a	*Rnaseh2c*
*Mutyh*	*Pold1*	Slx1b	*Rpa1*
*Neil1*	*Pold2*	Slx4	*Rpa2*
*Neil2*	*Pold3*	Telo2	*Rpa3*
*Neil3*	*Pold4*	Top3a	*Rpa4*
*Nthl1*	*Rfc1*	Top3b	*Ssbp1*
*Ogg1*	*Rfc2*	Ube2t	
*Parp1*	*Rfc3*	Usp1	
*Parp2*	*Rfc4*	Wdr48	
*Parp3*	*Rfc5*		
*Parp4*	*Rpa1*		
*Pcna*	*Rpa2*		
*Polb*	*Rpa3*		
*Pold1*	*Rpa4*		
*Pold2*	*Ssbp1*		
*Pold3*			
*Pold4*			
*Pole*			
*Pole2*			
*Smug1*			
*Tdg*			
*Ung*			
*Xrcc1*

**Table 3:** Non-DDR genes with variants detected in 2018 C57BL/6 J genome

Gene	No. variants	Gene	No. variants
Mroh2a	85	Crygb	3
C4b	53	Ly6c1	3
Kifc5b	43	Hsd3b5	3
Sirpb1c	37	Cyp3a41b	3
Gm8700	34	Vmn2r121	3
Naip1	33	Zfp979	3
Vmn2r117	25	Dux	3
Slc22a27	21	Gm14548	2
Cyp2a4	20	Gmps	2
Ceacam2	16	Zxdc	2
Thap1	16	Hnrnph2	2
Hjurp	15	Zfp975	2
Cyp3a44	15	Cyp3a11	2
Tdpoz1	15	Taf1b	2
Ang	14	Vmn2r89	2
Psg21	14	Gm3435	2
Fbxw14	13	Ubap2l	2
Vmn2r115	12	Fbxw24	2
Nlrp4f	11	Zfp985	2
Zfp982	11	Defa21	2
Caps2	10	Zfp456	2
Prb1	10	Cyp2b13	2
4930474N05Rik	10	Gm9758	2
Tulp4	10	Mrgpra2b	2
Vmn2r123	10	Zfp180	2
Ctla2b	9	Pfdn2	2
Obox1	9	Sp140	2
Cyp3a59	9	H2-Q6	2
Olfr102	8	Zfp873	1
Eef2	8	Rsph3b	1
Obox3	7	Ddx1	1
AY358078	6	2410141K09Rik	1
Phf5a	6	Bcl2a1b	1
Kng1	6	Gm3448	1
Srp54a	6	1110008L16Rik	1
Gckr	5	Pirb	1
H2-DMb2	5	Dmbt1	1
Aldh1a7	5	Gm14851	1
Gsta2	5	Vmn2r1	1
Vmn2r114	5	Cyp2b10	1
Ppcs	4	Zscan4f	1
Tmcc1	4	Naip5	1
Alms1	4	Speer4b	1
Cnot8	4	Speer4f1	1
Ces1c	4	A530032D15Rik	1
Cyp2b9	4	Ctdsp1	1
Atp6ap2	4	Gvin1	1
Ifi203	4	Ifi211	1
Speer4a	4	Pisd	1
Ly6a	4	4933416I08Rik	1
Mthfs	4	1110059E24Rik	1
Fbxw16	4	Slc22a21	1
C1ra,C1rb	4	Nit1	1
Jpt1	3	Cyp3a16	1
Atg4a	3	H2-Q7	1
Cdk9	3	4931408C20Rik	1
Prl7d1	3	Gm1979	1
1700049E17Rik1	3	Gm7827	1
Total		116	812

## Supplementary Information

**Additional file 1: Supplementary Table 1.** Total variants detected in 2018 C57BL/6J genome*

**Additional file 2: Supplementary Table 2.** Variants detected in 2018 C57BL/6J present in > 22 (50%) cases*

**Additional file 3: Supplementary Table 3.** A. Absence of coding variants in DDR genes by refering to mm7. B. Absence of coding variants in DDR genes by refering to mm10

**Additional file 4: Supplementary Table 4.** Variants in Non-DDR genes detected in C57BL/6J from 1998 to 2018

**Additional file 5: Supplementary Table 5.** Sanger-validated non-DDR variants in 2018 C57BL/6J

**Additional file 6: Supplementary Table 6.** Coding-variants in Eve6B differing from 1998 C57BL/6J genome*

## Data Availability

Exome data were deposited in NCBI SRA database (Accession no. PRJNA757631). All data generated from the study were included in the Supplementary Table S1, S2, S3, S4, S5 and S6.

## Abbreviations

DDR: DNA damage repair
HR: Homologous recombination
NHEJ: Non-homologous end joining
FA: Fanconi anemia pathway
BER: Base excision repair
NER: Nucleotide excision repair
MMR: Mismatch repair
SSA: Single-strand annealing
BRCA: BRCA1 and BRCA2
